# Anidulafungin compared with fluconazole for treatment of candidemia and other forms of invasive candidiasis caused by *Candida albicans*: a multivariate analysis of factors associated with improved outcome

**DOI:** 10.1186/1471-2334-11-261

**Published:** 2011-09-30

**Authors:** Annette C Reboli, Andrew F Shorr, Coleman Rotstein, Peter G Pappas, Daniel H Kett, Haran T Schlamm, Arlene L Reisman, Pinaki Biswas, Thomas J Walsh

**Affiliations:** 1 Division of Infectious Diseases, Cooper Medical School of Rowan University, 2 Aquarium Drive, Suite 305, Camden, NJ, 08103 USA; 2 Pulmonary Critical Care Section, Washington Hospital Center, Washington, DC, USA; 3 Division of Infectious Diseases, University of Toronto University Health Network, Toronto, ON, Canada; 4 Mycoses Study Group Division of Infectious Diseases, University of Alabama at Birmingham, Birmingham, AL, USA; 5 Department of Clinical Medicine, University of Miami Miller School of Medicine, Miami, FL, USA; 6 Pfizer Specialty Care (Anti-Infectives), Pfizer Inc, New York, NY, USA; 7 Pfizer Specialty Care (Statistics), Pfizer Inc, New York, NY, USA; 8 Pfizer Specialty Care (Statistics), Pfizer Inc, Collegeville, Pennsylvania, PA, USA; 9 Division of Infectious Diseases, Weill Cornell Medical College of Cornell University and New York Presbyterian Hospital, New York, NY, USA

**Keywords:** echinocandins, *Candida*, efficacy, safety, survival

## Abstract

**Background:**

*Candida albicans *is the most common cause of candidemia and other forms of invasive candidiasis. Systemic infections due to *C. albicans *exhibit good susceptibility to fluconazole and echinocandins. However, the echinocandin anidulafungin was recently demonstrated to be more effective than fluconazole for systemic *Candida *infections in a randomized, double-blind trial among 245 patients. In that trial, most infections were caused by *C. albicans*, and all respective isolates were susceptible to randomized study drug. We sought to better understand the factors associated with the enhanced efficacy of anidulafungin and hypothesized that intrinsic properties of the antifungal agents contributed to the treatment differences.

**Methods:**

Global responses at end of intravenous study treatment in patients with *C. albicans *infection were compared post-hoc. Multivariate logistic regression analyses were performed to predict response and to adjust for differences in independent baseline characteristics. Analyses focused on time to negative blood cultures, persistent infection at end of intravenous study treatment, and 6-week survival.

**Results:**

In total, 135 patients with *C. albicans *infections were identified. Among these, baseline APACHE II scores were similar between treatment arms. In these patients, global response was significantly better for anidulafungin than fluconazole (81.1% vs 62.3%; 95% confidence interval [CI] for difference, 3.7-33.9). After adjusting for baseline characteristics, the odds ratio for global response was 2.36 (95% CI, 1.06-5.25). Study treatment and APACHE II score were significant predictors of outcome. The most predictive logistic regression model found that the odds ratio for study treatment was 2.60 (95% CI, 1.14-5.91) in favor of anidulafungin, and the odds ratio for APACHE II score was 0.935 (95% CI, 0.885-0.987), with poorer responses associated with higher baseline APACHE II scores. Anidulafungin was associated with significantly faster clearance of blood cultures (log-rank *p *< 0.05) and significantly fewer persistent infections (2.7% vs 13.1%; *p *< 0.05). Survival through 6 weeks did not differ between treatment groups.

**Conclusions:**

In patients with *C. albicans *infection, anidulafungin was more effective than fluconazole, with more rapid clearance of positive blood cultures. This suggests that the fungicidal activity of echinocandins may have important clinical implications.

**Trial registration:**

ClinicalTrials.gov: NCT00058682

## Background

Despite the availability of new antifungal agents, systemic candidiasis (i.e., candidemia and other forms of invasive candidiasis) continue to contribute to excess morbidity, greater mortality, prolonged hospitalizations, and increased costs [1,2]. Epidemiological data demonstrate that the frequency of *Candida *infections is rising [3-6], along with an increase in the proportion of infections caused by non-*albicans Candida *species that are intrinsically resistant or variably susceptible to fluconazole [7-10]. However, *C. albicans *continues to be the most common *Candida *species isolated [6,11,12].

The vast majority of *C. albicans *isolates from bloodstream infections remain fully susceptible to fluconazole, which has been the treatment of choice for these infections in most settings [13]. However, updated IDSA guidelines now favor an echinocandin (anidulafungin, caspofungin, or micafungin) as first-line treatment for systemic candidiasis in moderately severe to severely ill patients or those with prior azole exposure [14]. Echinocandins have several potential advantages over fluconazole for the treatment of systemic candidiasis. They have a broader spectrum of activity (encompassing fluconazole-resistant *C. glabrata *and *C. krusei*) and exhibit potent fungicidal activity against most *Candida *species [15-17]. Echinocandins are highly active in vitro against *C. albicans*, with lower MICs than those of fluconazole [8,18]. While fluconazole and the echinocandins have similarly favorable safety profiles, the latter do not require dose adjustment in patients with renal insufficiency [19-21].

A phase III, randomized, double-blind study compared anidulafungin with fluconazole as primary treatment of systemic candidiasis in adult patients infected with any *Candida *species, except *C. krusei *[22]. Global response rates at the end of IV study treatment in mITT patients were significantly higher with anidulafungin (76%) than fluconazole (60%). The superiority of anidulafungin was questioned because of a potential center effect. However, a number of robust statistical tests all failed to show the presence of such an effect [22].

Of note, *C. albicans *was identified as the cause of infection in the majority of cases (62%) and the difference in global responses among this clinically important subgroup was remarkable: 81% with anidulafungin versus 62% with fluconazole (*p *= 0.02) [22]. Since *C. albicans *isolates were almost uniformly susceptible to fluconazole [22], the excess failures in the fluconazole group could not be attributed to antifungal resistance. However, due to the lack of a multivariable analysis, it was unclear whether these differences were potentially related to unknown host factors. We therefore conducted a post-hoc analysis using data collected from that trial, in order to explore the factors associated with the better global response of anidulafungin, specifically in patients with *C. albicans *infections.

## Methods

### Study Design

The design of the original randomized clinical trial in patients with confirmed candidemia or other forms of invasive candidiasis has been described in detail previously [22]. Enrolled patients were randomized to receive blinded IV treatment with either anidulafungin or fluconazole; after ≥ 10 days, the antifungal regimen could be changed to open-label oral fluconazole. The primary endpoint was global response at end of IV study treatment in the mITT population. Other comparisons included time to negative blood culture, rates of persistent infection at end of IV study treatment (as assessed by the investigator based on available clinical and microbiologic data), and 6-week survival.

For the purposes of this post-hoc analysis, the study database was reviewed to identify all patients with systemic candidiasis caused by *C. albicans *only. Patients with non-*albicans Candida *infections and mixed infections (*C. albicans *and another concurrent pathogen) at baseline were excluded from all subsequent analyses.

### Microbiology

Screening blood cultures were obtained for all patients; those cultures obtained > 24 hours before study entry were repeated at baseline. Per the study protocol, blood cultures were also to be obtained on Day 3, 7, and subsequently every 3 days until negative while on study medication. Additional blood cultures could be obtained when clinically indicated, at the investigator's discretion. For patients with microbiologic evidence of infection from sites other than blood, culture from the same site was repeated as clinically indicated. All *Candida *isolates were submitted to a reference laboratory, where MICs were determined according to standard methods current at the time of the study [23,24].

### Statistical Analyses

The following analyses were conducted using data collected from all patients with *C. albicans *infection-only enrolled in the original clinical trial:

• In order to determine whether treatment arms differed significantly in terms of independent baseline characteristics (see Table 1 for complete list, including risk factors for systemic candidiasis), these characteristics were compared by univariate analysis using the chi-square test or Fisher's exact test, as appropriate. Exceptions were age and APACHE II score, which were compared using the *t*-test. The same methods were also used to identify all baseline characteristics that differed between groups at the *p *≤ 0.2 level.

**Table 1:** Baseline characteristics of patients infected with *C. albicans *only.

Characteristic	Anidulafungin (n = 74)	Fluconazole (n = 61)	*p*
Sex, n (%)			0.858
Male	39 (52.7)	34 (55.7)	
Female	35 (47.3)	27 (44.3)	
Age (years)^a^			0.097^b^
Mean ± SD	54.6 ± 17.9	59.7 ± 17.1	
Range	16-89	24-91	
Race or ethnic group, n (%)			0.823
White	59 (79.7)	47 (77.0)	
Black	7 (9.5)	8 (13.1)	
Other	8 (10.9)	6 (9.8)	
Risk factors for *Candida *infection, n (%)			
Central venous catheter	54 (73.0)	47 (77.0)	0.691
Catheter removed within 24 h of study entry	70 (94.6)	56 (91.8)	0.731
Broad-spectrum antibiotics^a^	43 (58.1)	44 (72.1)	0.106
Recent surgery	32 (43.2)	29 (47.5)	0.728
Recent hyperalimentation	17 (23.0)	12 (19.7)	0.678
Underlying malignancy	18 (24.3)	18 (29.5)	0.560
Immunosuppressive therapy	11 (14.9)	12 (19.7)	0.497
APACHE II score, n (%)			0.605^b^
≤ 20	60 (81.1)	52 (85.2)	
> 20	14 (18.9)	9 (14.8)	
Mean ± SD (median)	13.7 ± 7.8 (12)	14.3 ± 6.4 (14)	
Range	2-37	3-30	
Absolute neutrophil count, n (%)^a^			0.175
> 500/mm^3^	73 (98.6)	57 (93.4)	
≤ 500/mm^3^	1 (1.4)	4 (6.6)	
Site of infection, n (%)			0.343
Blood only	67 (90.5)	51 (83.6)	
Deep tissue infection	7 (9.5)	10 (16.4)	

^a ^Baseline variables significant at the *p *≤ 0.20 level.

^b ^*p *value calculated using a *t*-test, assuming continuous distribution of this variable.

• In order to compare global response between treatment arms after adjusting for baseline factors, all those baseline characteristics that differed at the *p *≤ 0.2 level were placed into a multivariate logistic regression model with global treatment success at end of IV study treatment as the response variable. Treatment was retained in the model and all variables were assessed for co-linearity.

• Thereafter, a best logistic regression model was selected based on the Akaike Information Criterion [25], in order to identify individual baseline variables that significantly predicted treatment success at end of IV study treatment. Adjusted odds ratios for treatment and other variables were calculated, together with their respective 95% CIs. Since this was an exploratory post-hoc study, no adjustment for multiple comparisons was made.

• In order to account for center-to-center variability, this final model was then adjusted to also incorporate center as a random effect.

To compare the time to negative blood cultures, all patients with a positive blood culture within 24 hours of study entry (Day 1) were identified. Results of blood cultures obtained per study protocol, and as clinically indicated, were used in this analysis. The time in days to the first documented sustained negative blood culture was incorporated into a Kaplan-Meier analysis, and differences between treatments were assessed using both the log-rank test and the Wilcoxon test. Patients with invasive candidiasis confirmed through positive deep-tissue culture only (i.e. without concomitant isolation of *Candida *from the bloodstream) were excluded from this time-to-event analysis, since tissue cultures were not obtained according to the same pre-specified schedule as blood cultures. The incidence of persistent *Candida *infection was compared using chi-square analysis. Kaplan-Meier analyses of survival were generated and the difference in survival between treatments at 6 weeks from study entry was assessed using the log-rank test. All statistical analyses were conducted using SAS version 8.2 (SAS Institute Inc., Cary, NC).

## Results

### Baseline Characteristics

The overall mITT population of 245 patients included 135 patients with *C. albicans *infection only; 74 of these had been randomized to anidulafungin and 61 to fluconazole. Seventeen (12.6%) patients were enrolled at a single study site; 28 sites (out of a total of 36) each enrolled < 5% of all mITT patients with *C. albicans*. Most presented with candidemia only (90.5% vs 83.6% of patients treated with anidulafungin and fluconazole, respectively; *p *= 0.34 by chi-squared test); the remaining patients had *C. albicans *isolated from other normally sterile sites (including peritoneal fluid/abdominal abscess or pleural fluid), with or without concomitant isolation from the bloodstream. Baseline characteristics for patients with *C. albicans *were similar to those of the overall patient population [22]. There were no significant differences between the two treatment groups in baseline demographic or clinical characteristics, including age, sex, race, APACHE II score, neutropenia, and other possible risk factors for invasive *Candida *infection (Table 1). Central venous catheters present at baseline were removed from all but nine patients (four in the anidulafungin and five in the fluconazole group).

### Susceptibility of *C. Albicans *Isolates

Over 90% of blood cultures were collected at those time points specified by the study protocol (± 1 day). The median number of blood cultures collected per patient was 16 in each arm. The *C. albicans *isolates tested were uniformly susceptible to anidulafungin (MIC range, ≤ 0.002 to 0.03 μg/ml). All but one of the baseline *C. albicans *isolates were susceptible to fluconazole (MIC range, ≤ 0.06 to > 128 μg/ml). Of note, the single patient with a fluconazole-resistant *C. albicans *isolate at baseline (fluconazole MIC > 128 μg/ml) was randomized to receive anidulafungin.

### Efficacy

#### Global Response

**Figure 1:**
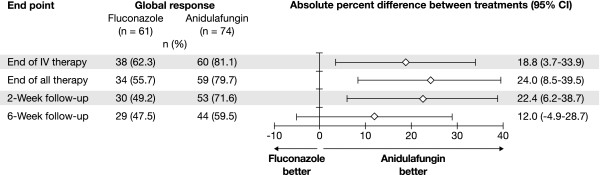
**Global response rates at prespecified time points**.

The investigator-assessed global response rate at end of IV study treatment was higher in patients with *C. albicans *infections treated with anidulafungin compared to fluconazole: 81.1% versus 62.3% (difference 18.8%; 95% CI, 3.7-33.9). The difference was statistically significant at end of IV study treatment (*p *= 0.02), and remained significant at the end of all treatment and at the 2-week follow-up (Figure 1). In the few patients with central venous catheters at baseline who did not have these removed, global response rates at end of IV study treatment were 3/4 for anidulafungin and 3/5 for fluconazole.

#### Univariate Analyses and Multivariate Logistic Regression

There were no significant (*p *≤ 0.05) imbalances in any baseline clinical or demographic characteristics between the two treatment groups (Table 1). The significant difference in global response at end of IV study treatment between the two treatment groups observed in the original clinical trial for patients with *C. albicans *[22] was maintained in the multivariate logistic regression model after adjustment for all baseline variables that differed between groups at the *p *≤ 0.2 level (adjusted odds ratio, 2.36; 95% CI, 1.06-5.25). Specifically, these baseline variables were age, use of broad-spectrum antibiotics, and absolute neutrophil count. In further analyses using the Akaike Information Criterion to select a best model, study treatment and APACHE II score (as a continuous variable) were identified as significant and independent predictors of global response at end of IV study treatment in patients with invasive *C. albicans *infection. From that analysis, after accounting for center as a random effect, the odds ratio for study treatment was 2.60 (95% CI, 1.14-5.91) in favor of anidulafungin, and the odds ratio for APACHE II score was 0.935 (95% CI, 0.885-0.987), with poorer responses associated with higher baseline APACHE II scores. Additional analyses adjusting for a potential center effect, i.e., grouping the largest-enrolling center versus the rest and grouping all centers that enrolled ≥ 5% of patients versus the rest, resulted in very similar odds ratios (2.57 and 2.67, respectively) and 95% CIs for the treatment effect.

#### Time to Negative Blood Culture

**Figure 2:**
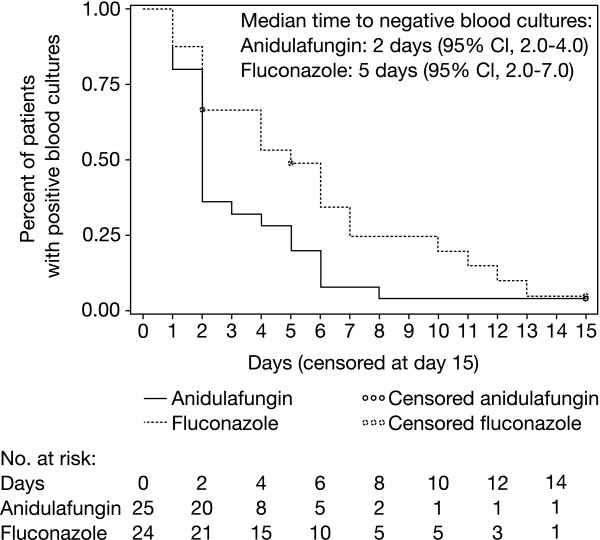
**Kaplan-Meier estimates of time to first negative blood culture**. Analyses included patients with positive baseline cultures for *C. albicans *(n = 49)

Forty-nine patients had positive blood cultures for *C. albicans *within 24 hours of study entry. These positive blood cultures cleared significantly more rapidly in patients treated with anidulafungin than those on fluconazole (Figure 2). In a Kaplan-Meier analysis, the time to negative blood culture was significantly shorter for anidulafungin compared with fluconazole (log-rank *p *< 0.05); median times to negative blood culture were 2 and 5 days, respectively. Persistent infection was reported in two patients (2.7%) in the anidulafungin group compared with eight (13.1%) in the fluconazole group (*p *< 0.05).

#### Survival

The proportion of patients who died during the 6-week period from study entry was 20.3% (15/74) in the anidulafungin arm and 21.3% (13/61) in the fluconazole arm; the Kaplan-Meier estimates of survival at 6 weeks were not significantly different between treatment groups (*p *= 0.842 by log-rank test). However, fewer deaths occurred within 24 hours of end of treatment with anidulafungin than with fluconazole (4 vs 13; *p *= 0.01 by chi-square test).

#### Treatment Duration, Discontinuations, and Adverse Events

Both study drugs were well tolerated and the respective safety profiles in patients with *C. albicans *infection only were similar to those in the overall study population [22]. The mean duration of IV treatment was 13.8 days (range, 1-33 days) for anidulafungin and 12.6 days (range, 2-33 days) for fluconazole. The proportion of patients who received open-label fluconazole after end of IV study treatment was similar in both groups: 23/74 for anidulafungin (31.1%) and 21/61 (34.4%) for fluconazole.

The reasons for treatment failure and for discontinuation of study treatment are summarized in Table 2. Treatment discontinuations were more common in the fluconazole arm than in the anidulafungin arm, primarily because of worsening clinical status. Discontinuations due to treatment-related adverse events were reported in one patient receiving anidulafungin (elevated liver enzymes) and four patients receiving fluconazole (worsening candidemia [n = 1], elevated liver enzymes [n = 2], and rash [n = 1]).

**Table 2:** Reasons for failure of and withdrawal from study medication.

	Anidulafungin	Fluconazole
Observed failure^a^	4	13
Clinical success and microbiologic failure^b^	0	1
Clinical failure and microbiologic success	1	4
Clinical failure and microbiologic failure^b^	3	7
Clinically indeterminate and microbiologic failure^b^	0	1
Indeterminate response^c^	10	10
Withdrawal from study medication	12	21
Due to adverse event^d^	7	7
Due to withdrawal of consent	2	1
At investigator's discretion	1	3
Worsening clinical status	2	9
Death within 24 hours of end of IV study treatment^b^	4	7
Receipt of < 3 doses of study medication	1	1

^a ^Observed failure was defined as treatment failure declared by the investigator to be clinical failure, microbiologic failure (i.e. positive findings on culture), or both. In these patients mean time to failure was 16.8 days for anidulafungin and 8.9 days for fluconazole.

^b ^Among those in whom microbiologic failure occurred, two of three patients in the anidulafungin group and eight of nine in the fluconazole group had persistent infection. The other two patients in whom microbiologic failure occurred had superinfections.

^c ^For the primary efficacy analysis, indeterminate response was treated as failure of global response. Patients could be considered to have an indeterminate response for more than one reason.

^d ^In the anidulafungin group, adverse events leading to discontinuation included sepsis (2), renal failure (1), elevated liver enzymes (1), multisystem organ failure (1), cardiac failure (1), and hypoglycemia (1). In the fluconazole group, adverse events leading to discontinuation included renal failure (3), elevated liver enzymes (2), cancer (1), and multisystem organ failure (1).

## Discussion

This study represents the first clinical analysis of differential response in patients with systemic candidiasis caused by *C. albicans *to an echinocandin versus fluconazole, conducted post-hoc in the relevant subpopulation from a prospective clinical trial. The significance of this analysis is that it enabled us to directly compare the efficacy of a fungicidal with that of a fungistatic drug in patients with *C. albicans *infections, without potential confounding by differences in susceptibility. Despite all isolates of *C. albicans *being susceptible to the antifungal agent received by each patient, response to anidulafungin was significantly greater than to fluconazole. Logistic regression analysis, using the Akaike Information Criterion methodology, demonstrated that study drug and baseline APACHE II score were the principal independent variables determining this outcome, reflecting pharmacological and host factors, respectively. Study drug remained a significant predictor of treatment success even after adjustment for APACHE II scores and center variability, with an estimated OR of 2.6. While there was notable variation in this odds ratio, in part due to the relatively small sample size, it remained significant from a statistical perspective and should therefore be considered important. These results suggest a pharmacological benefit of anidulafungin in the treatment of systemic candidiasis due to *C. albicans*, since no other factor, such as potential baseline imbalances and center variability, was shown to have impacted the outcome.

Understanding the factors determining clinical outcomes in patients with *C. albicans *infections was important, since treatment differences among this most common subpopulation in turn drove the treatment differences observed in the overall clinical trial [22]. Among patients infected with *C. albicans*, anidulafungin resulted in better global response at end of IV study treatment, at the end of all treatment, and at the 2-week follow-up. Moreover, patients treated with anidulafungin also had more rapid eradication of yeast from the blood, as well as lower rates of persistent *C. albicans *infection.

Whether the better response of anidulafungin in the subpopulation of patients with systemic candidiasis caused by *C. albicans *emerged as the result of an imbalance in characteristics between the two treatment arms was not evident from the previously available data. However, the present analyses show that the efficacy differences were not due to imbalances in baseline clinical or demographic characteristics between treatment arms. There was also no imbalance in the proportion of patients whose central venous catheters remained in place during the study; this factor is considered important, because removal or replacement of central venous catheters may lead to better outcomes [26,27]. Moreover, the lower response to fluconazole could also not be explained by in vitro resistance. Finally, first-line therapy with anidulafungin was identified as a significant and independent predictor of successful treatment outcome and remained so after adjustment for APACHE II score.

Even though we did not specifically test fungicidality, the recognized fungicidal properties of the echinocandin may have contributed to this outcome. In fact, the more rapid clearance of blood cultures and greater patient survival during the early phase of therapy are consistent with the pharmacodynamic properties of a fungicidal agent [15,16,28-30]. Similar results have also been observed in pre-clinical studies. For instance, a neutropenic murine model of disseminated *C. albicans *infection demonstrated that anidulafungin yielded significantly greater reduction of fungal burden in the kidneys than fluconazole [31] and, in a neutropenic rabbit model of systemic candidiasis, anidulafungin cleared *C. albicans *from tissues more effectively than fluconazole [32]. Organism-mediated tissue injury appears to be an intrinsic component of *C. albicans *pathogenesis [33-37], and failure to quickly control candidemia can lead to disseminated infection and poor outcomes [38,39]. Considered together, these results suggest that the enhanced fungicidal activity of echinocandins may have an impact on treatment outcomes in invasive *C. albicans *infections.

Although there was a clear clinical benefit with anidulafungin, this did not translate into a difference in long-term survival. We can merely speculate why this may have occurred. It is possible that later deaths were attributable to underlying illnesses rather than to systemic candidiasis. In a matched case-controlled study among candidemia-exposed and candidemia-unexposed patients, slightly less than half of the overall mortality was caused by candidemia [2]. Another possible explanation is that optimal management of patients with invasive *Candida *infections includes other interventions besides antifungal therapy, and these factors were not controlled for in our study. Also, the clinical trial may have selected for patients with better prognoses, thus making it more difficult to demonstrate a survival benefit.

Our study has several potential limitations. Although the comparison of global response in patients with *C. albicans *infection was planned *a priori*, the analyses described in this manuscript were performed post-hoc in a subset of patients, albeit using prospectively collected data from a double-blind, randomized clinical trial. Owing to the post-hoc nature of these analyses, the two treatment groups did not have the same sample size, which was not chosen to be statistically powered. However, this slight disproportion should not impact our overall results, as all relevant differences in baseline factors were adjusted for using multivariate logistic regression; the odds ratio for the treatment effect is thus corrected for any baseline imbalances. Of note, despite incorporating a relatively large number of baseline variables into the multivariate analyses, there may have been unidentified confounding variables with a potentially significant impact on response. Since no adjustment was made for multiplicity, the results should be interpreted with some caution. Another potential limitation is the lack of daily blood culture collection during the original trial, which precluded an accurate determination of the exact time to negative blood culture; the corresponding analysis should thus be interpreted with some caution. This shortcoming is somewhat compensated for by the fact that the vast majority of blood cultures were collected on time points (± 1 day) prespecified by the study protocol. Finally, extrapolation of our results to all patients with systemic candidiasis due to *C. albicans *may not be appropriate, since we focused on patients with *C. albicans *only and excluded those with mixed (*albicans *and non-*albicans*) infections.

## Conclusions

This analysis based on prospectively collected data from a pivotal clinical trial confirms that anidulafungin had better efficacy than fluconazole for the treatment of candidemia or invasive candidiasis due to *C. albicans*. In these patients with systemic candidiasis caused by fluconazole-susceptible *C. albicans*, anidulafungin was more effective than fluconazole in terms of better global response, faster clearance of *Candida *from the bloodstream, and fewer persistent infections.

## Abbreviations

APACHE: Acute Physiology and Chronic Health Evaluation; CI: confidence interval; IDSA: Infectious Diseases Society of America; IV: intravenous; MICs: minimum inhibitory concentrations; mITT: modified intention-to-treat; SD: standard deviation.

## Competing interests

Potential conflicts of interest: ACR has received clinical research grant support from Merck and Pfizer, has been a consultant for Merck, Astellas, and Pfizer, and has been a lecturer for Pfizer and Merck. AFS has served as a consultant to, speaker for, and/or has received research support from Astellas, Merck, and Pfizer. CR has received grant/research support from Astellas, Basilea, Johnson & Johnson, Merck, and Pfizer; has been a consultant to Astellas, Bayer, iCo, Merck, and Pfizer; and has served on Speakers' Bureaus for Astellas, Bayer, Johnson & Johnson, Merck, and Pfizer. PGP has received grant/research support from Merck, Pfizer, and Astellas, and has been an ad hoc advisor to Novartis, Merck, Astellas and Pfizer. DHK has received research support from Pfizer, Astellas, and Akers Bioscience; has been a consultant to Pfizer and Astellas; and has served as a speaker for Pfizer, Astellas, and GlaxoSmithKline. TJW has served as consultant to Trius, Novartis, Vestagen, Sigma Tau, and iCo. HTS, ALR, and PB are full-time employees of Pfizer Inc.

## Authors' contributions

ACR, CR, PGP, DHK and TJW participated in the original clinical trial. ALR and PB conducted the statistical analyses described in this paper. ACR, AFS, HTS and TJW interpreted the statistical analyses, with subsequent input from CR, PGP, and DHK. ACR and HTS wrote the first draft of the manuscript. All authors critically revised the manuscript for important intellectual content and have read and approved the final version.

## Pre-publication history

The pre-publication history for this paper can be accessed here:

http://www.biomedcentral.com/1471-2334/11/261/prepub
